# Functional evaluation of GUCY1A3 mutations associated with myocardial infarction risk

**DOI:** 10.1186/2050-6511-16-S1-A100

**Published:** 2015-09-02

**Authors:** Jana Wobst, Tan An Dang, Thorsten Kessler, Simon von Ameln, Stephanie Tennstedt, Christian Hengstenberg, Jeanette Erdmann, Heribert Schunkert

**Affiliations:** 1 Klinik für Herz-und Kreislauferkrankungen, Deutsches Herzzentrum München, Technische Universtität München, Munich, Germany; 2 Institut für Integrative und Experimentelle Genomik, Universität zu Lübeck, Germany; 3 Deutsches Zentrum für Herz-Kreislauf-Forschung (DZHK) e.V., partner site Munich Heart Alliance (MHA), Munich, Germany; 4 Deutsches Zentrum für Herz-Kreislauf-Forschung (DZHK) e.V., partner site Hamburg/Kiel/Lübeck, Lübeck, Germany

## 

Myocardial infarction (MI) is the main complication of coronary artery disease (CAD). Recently, a locus tagging the *GUCY1A3* gene has been shown to be genome-wide significantly associated with CAD [1]. *GUCY1A3* encodes for the α_1_-subunit of the soluble guanylyl cyclase (sGC) which consists of α_1_- and β_1_-subunits and catalyzes the production of cGMP upon stimulation with nitric oxide (NO). cGMP acts a second messenger that mediates diverse cellular functions, e.g. smooth muscle relaxation and inhibition of platelet aggregation. Using whole-exome sequencing, our group also identified nine rare variants in the coding sequence of *GUCY1A3* [2]. Two of these variants were found in two extended families with a high prevalence of premature CAD/MI. Seven further rare variants were found in 252 young MI patients. In this study, we aimed to investigate the functional implication of these rare variants found in CAD/MI patients (Table 1) regarding protein level, dimerization capability and enzymatic activity.

**Table 1:** Rare variants of sGC α1 subunit found in MI patients:

Variant	Identified in	Predicted effect on protein function		
		
		PolyPhen-2	SIFT	SNAP
p.Leu163Phefs*24	MI family	frameshift	-	-
p.Lys53Glu	252 young MI cases	possibly damaging damaging	tolerated	non-neutral
p.Thr64Ala	252 young MI cases	benign	tolerated	neutral
p.Thr229Met	252 young MI cases	possibly damaging	tolerated	non-neutral
p.Ser478Gly	252 young MI cases	benign	tolerated	neutral
p.Val587Ile	252 young MI cases	benign	tolerated	neutral
p.Gly573Arg	MI family	probably damaging	affect protein function	non-neutral
p.Cys610Tyr	252 young MI cases	probably damaging	tolerated	neutral
p.Ile571Val	252 young MI cases	possibly damaging	affect protein function	neutral

Two of the investigated α_1_ variants exhibited significantly decreased protein levels compared to wild type α_1_. The amount of β_1_ correlated with those of α_1_ in all cases. All α_1_ variants, except for p.Leu163Phefs*24, still dimerized with the β_1_ subunit, as shown by co-immunoprecipitation. Using radioimmunoassay three of the rare variants demonstrated significantly decreased cGMP amounts at every time point tested (0.5/1/2 min). The activity only in part correlated with the observed protein levels pointing to an effect of the tested variants on enzymatic activity. As we have shown that loss of function-mutations in *GUCY1A3* may lead to CAD/MI [2], decreased enzymatic activity might also increase risk. Future studies focus on mRNA abundance and protein degradation to uncover the reason for attenuated activity of the respective variants.
